# Single-cell mechanics and calcium signalling in organotypic slices of human myometrium^☆^

**DOI:** 10.1016/j.jbiomech.2015.01.046

**Published:** 2015-06-25

**Authors:** Fiona C. Loftus, Magnus J.E. Richardson, Anatoly Shmygol

**Affiliations:** a Warwick Systems Biology Centre, University of Warwick, Coventry, UK; b Division of Translational and Systems Medicine, Warwick Medical School, University of Warwick, Coventry, UK; c Warwick Systems Biology Doctoral Training Centre, University of Warwick, Coventry, UK

**Keywords:** Myometrium, Calcium signalling, Motion artifacts correction, Oxytocin

## Abstract

Elucidation of cellular mechanisms regulating myometrial contractility is crucial for improvement in management of many obstetric abnormalities, such as premature delivery, uterine dystocia and post-partum haemorrhage. Myometrial contractions are triggered by periodic synchronous rises in intracellular calcium concentration ([Ca^2+^]_i_) elicited by spontaneously generated action potentials propagating throughout the entire myometrium. During labour, hormones like oxytocin and prostaglandins potentiate uterine contractions by increasing their duration, strength and frequency. The most informative approach to studying the mechanisms underlying hormonal modulation of uterine contractility is to record [Ca^2+^]_i_ responses to hormones in intact myometrial samples that have not been subjected to enzymatic treatment for cell isolation or cell culture conditions. However, the spatio-temporal resolution of such recording is limited due to the motion artifacts occurring in contracting tissue. Here we describe the application of our newly developed motion correction algorithm to investigate the [Ca^2+^]_i_ dynamics in control and oxytocin stimulated slices of human myometrium on a cellular level. We present evidence that oxytocin induces asynchronous [Ca^2+^]_i_ oscillations in individual myocytes within intact myometrium which are similar to those observed in cultured cells. The oscillations occur between synchronous action potential-driven [Ca^2+^]_i_ transients but appear to be unrelated to contractions. Furthermore, the oxytocin-triggered [Ca^2+^]_i_ oscillations wane within 30–50 min of hormone application, while the action potential induced [Ca^2+^]_i_ transients remain augmented. We conclude that oxytocin-induced [Ca^2+^]_i_ oscillations are not relevant to the acute regulation of myometrial contractility but may play a role in longer-term regulatory processes, for example, by triggering gene expression.

## 1 Introduction

Successful childbirth depends on precisely timed and coordinated uterine contractions. These contractions are triggered by periodic synchronous rises in [Ca^2+^]_i_ elicited by propagating action potentials (Parkington et al., 1999). The rise in [Ca^2+^]_i_ triggers calmodulin-dependent phosphorylation of the regulatory light chains of myosin, leading to the generation of mechanical force via repetitive attachments, power strokes and detachments of cross-bridges between the actin and myosin filaments (cross-bridge cycling). The rate of cross-bridge cycling is controlled by the relative equilibrium of activity between myosin light-chain kinase that phosphorylates the regulatory myosin light chains and myosin light-chain phosphatase that dephosphorylates them (Word et al., 1994). The resulting excitation–contraction–relaxation cycle is further modulated by hormonal signals. That is, the amplitude, duration and frequency of uterine contractions are increased during labour by oxytocin and prostaglandin F2α. Unsurprisingly, a lot of effort has been spent on investigation of the molecular and cellular mechanisms of action of uterotonic hormones. The modulatory effects of oxytocin and prostaglandins on the relationship between [Ca^2+^]_i_ and force in human myometrium has been investigated using microfluorimetry combined with mechanography in strips loaded with ratiometric Ca^2+^ sensitive dyes Fura-2 (Fomin et al., 2006, 2009; McKillen et al., 1999; Word et al., 1994) or Indo-1 (Longbottom et al., 2000; Luckas et al., 2000). A major drawback of Ca^2+^ microfluorimetry is that it gives a spatially averaged signal with no information on cellular distribution of [Ca^2+^]_i_. Details of cellular and sub-cellular [Ca^2+^]_i_ responses to uterotonic hormones have been studied using digital imaging of cultured uterine myocytes loaded with Ca^2+^-sensitive indicators (Fu et al., 2000; Young and Hession, 1996; Young and Zhang, 2001). These studies have revealed that application of oxytocin produces repetitive [Ca^2+^]_i_ oscillations and [Ca^2+^]_i_ waves propagating within the cytoplasm of individual cells and also between neighbouring myocytes (Young and Zhang, 2001). However, it remains unclear how [Ca^2+^]_i_ oscillations relate to the oxytocin-induced augmentation of phasic myometrial contractions in intact myometrium. Recording the time course of [Ca^2+^]_i_ in individual cells within spontaneously contracting and oxytocin-stimulated multicellular preparations dissected from intact myometrium could answer this question. However, the spatio-temporal resolution of [Ca^2+^]_i_ dynamics is limited due to the motion artifacts occurring in contracting tissue (Bru-Mercier et al., 2012). Recently, we developed an image processing algorithm for off-line correction of motion artifacts that allows extraction of [Ca^2+^]_i_ dynamics on a cellular level within multicellular tissue slices (Loftus et al., 2014). Here, we used this processing algorithm to investigate the [Ca^2+^]_i_ dynamics in control and oxytocin stimulated slices of human myometrium on a cellular level. For the first time, we present evidence that oxytocin induces asynchronous [Ca^2+^]_i_ oscillations in individual myocytes within intact myometrium which are similar to those observed in cultured cells. The oscillations occur between synchronous action potential-driven [Ca^2+^]_i_ transients but appear to be unrelated to contractions.

## 2 Methods

### 2.1 Tissue procurement and slice preparation

Myometrial biopsy specimens were obtained with informed written consent (information leaflet Ref: PTL220705) and approval from the Local Ethics Committee at University Hospital Coventry and Warwickshire (REC-05/Q2802/107) from term-pregnant women (≥37 weeks gestation) undergoing elective caesarean section before the onset of labour.

Myometrial specimens used in this study were obtained from 12 different patients. Samples were collected into sterile 25 ml plastic tubes filled with cold Krebs solution, placed on ice and delivered to the laboratory within 20–30 min. On arrival, each biopsy specimen was trimmed under a stereo microscope into a strip of approximately 1.0×0.5×0.3 cm^3^. The strip was then ligatured with braided 2-0 Mersilk suture (Ethicon, Inc., UK) at both ends before being stretched and fixed to the base of a stainless steel tissue holder using cyanoacrylate glue. From this, 200 μm-thick slices were cut using an oscillating vibroslicer (Integraslice 7550 PSDS, Campden Instruments, UK) in oxygenated ice-cold Krebs solution. Slicing was performed with razor blades at an oscillating speed of 86 Hz with lateral amplitude of 1 mm and an advance speed of 0.10–0.20 mm s^−1^. First cuts and the glued base of the strip were discarded. Each slice was then separated by cutting the extremity of the slice using fine dissecting scissors and transferred into Krebs solution. After 1 h incubation at room temperature for equilibration and recovery and 30 min loading with Fluo-4/AM (see below) the slices were used in experiments within 4–6 h.

### 2.2 Confocal imaging of [Ca^2+^]_i_

For [Ca^2+^]_i_ recording, slices were incubated for 30 min at 37 °C in Krebs solution containing 13 μM Fluo-4/AM (Invitrogen, UK). Non-ionic detergent Pluronic F127 (0.025%, w/v) was included to aid the dye loading. This protocol produced homogeneous staining of cells throughout the slice and gave better tissue viability compared to loading at room temperature, which required up to 4 h incubation to achieve the same intensity of Fluo-4 signal. The loaded slice was placed in a glass-bottomed Petri dish and weighted down with a 250 mg slice grid (HSG-5, ALA Scientific, USA). Since no stretch was applied to slices used in these experiments, the resting tension was estimated to be zero mN. The dish was then secured in a spring-loaded holder in a temperature-controlled environmental chamber on the stage of an inverted microscope (Axiovert 200 M) equipped with an LSM 510 META confocal scanner (Karl Zeiss, UK) and superfused with pre-warmed (35 °C) Krebs solution at a flow rate of 2 ml min^−1^ for 30–40 min until stable spontaneous contractions developed. Slices that failed to develop spontaneous activity within this timeframe were excluded.

Confocal imaging of Fluo-4 loaded slices was achieved by scanning a 488 nm wavelength laser beam focussed into a diffraction-limited spot via a Fluar 5×/0.25NA objective lens and recording fluorescence through a band-pass filter (505–530 nm) by a photomultiplier tube with a pinhole in front of it. The pinhole diameter was set to 2 Airy units to reject most of the out-of-focus fluorescence and to maximise the throughput of light originating from the focal plane. The use of low-magnification objective lens allowed us to record from a large area measuring 1.8 mm×0.9 mm. Superior light-gathering ability of the high numerical aperture objective lens used in our study was crucial for ensuring a good signal to noise ratio even at lowest intensity of laser illumination. Keeping the intensity of illumination at low level was critical for preventing the photobleaching during prolonged recordings. The image acquisition was controlled by Zeiss LSM v4.0 software. Time series of up to 8000 frames were collected and stored on a hard drive for off-line analysis.

### 2.3 Image processing and data analysis

The LSM files were imported into ImageJ (NIH, http://imagej.nih.gov/ij/) using the LSM Toolbox plug-in (http://imagejdocu.tudor.lu/doku.php?id=plugin:inputoutput:lsmtoolbox:start).

Examination of recorded time series in ImageJ revealed that substantial motion artifacts were present. To remove these artifacts, the image sequences were imported into MATLAB (Mathworks, Natick, MA, USA) and processed using our motion-correction algorithm. A detailed description of the motion correction procedure has been given elsewhere (Loftus et al., 2014). Briefly, the main steps of the algorithm comprise: (i) landmark identification by band-pass filtering the image to emphasise the bright blobs always present in the images as potential landmarks; (ii) tracking the motion of each landmark between frames for the entire image stack and removing outlier landmarks; and (iii) extrapolating the motion of landmarks to all neighbouring pixels to yield a complete description of the tissue motion. The output of the motion-correction algorithm was a sequence of tiff files with motion artifacts removed. These were further analysed in ImageJ to extract single-cell [Ca^2+^]_i_ dynamics. Regions of interest (ROI) encompassing individual cells were drawn by hand and the ROI Manager in ImageJ was used to create a list of up to 25 ROIs. This list was then applied to the motion corrected image stack and the Multi Measure function in the ROI Manager was used to extract the intensity profiles over time for each ROI. The ROI intensity profiles were imported into Origin 9.1 (OriginLab Corporation, USA) for further processing, graphing and statistical analysis. The intensity vs. time profile from each ROI was plotted in Origin and the baseline connecting the lowest values of fluorescence between successive contractions in each trace was drawn. Each trace was then normalised to its corresponding baseline to yield a photobleaching-corrected self-ratio trace (*F*/*F*_0_) with resting [Ca^2+^]_i_ corresponding to *F*/*F*_0_=1 and upward deflections corresponding to [Ca^2+^]_i_ increase.

To monitor the time course of contraction, a larger ROI was placed over the moving edge of the slice in the corresponding uncorrected image sequence. The mean intensity obtained from this ROI is proportional to the displacement of the slice as it moves in and out of the ROI during contraction–relaxation cycle. In contrast to other traces, the displacement trace was normalised to the highest values of intensity between contractions, when the slice was fully relaxed. The relaxed state therefore corresponds to *F*/*F*_0_=1 and downward deflections follow the time course of contractions.

## 3 Results

**Fig. 1:**
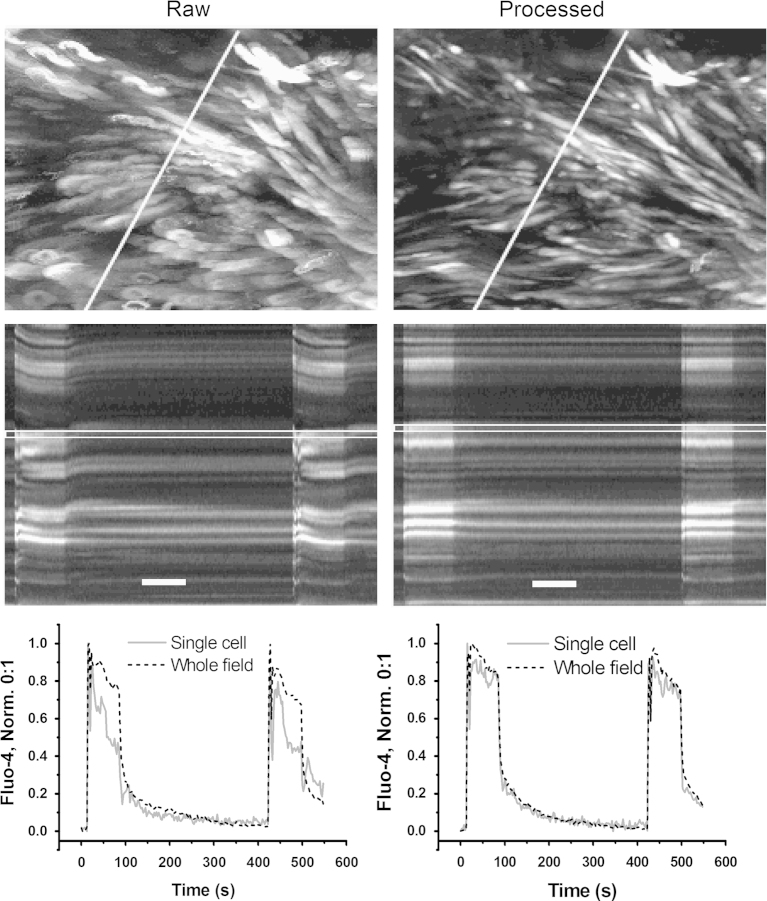
Illustration of the motion artifacts removal by computational image processing. Left-hand side panels illustrate raw data, right-hand side panels – data extracted from the motion corrected sequence. From top to bottom: maximum intensity projections of image sequence containing two contraction–relaxation cycles; pseudo line-scan images obtained by re-slicing along the line in top panel; and superposition of normalised [Ca^2+^]_i_ traces from a single cell indicated by a rectangle in the middle panel (solid line) and from the entire field (dashed line). Note almost complete removal of motion artifacts after image processing on the right. White scale-bars in the middle panel correspond to 120 s.

Fig. 1 illustrates the performance of our motion correction procedure. The length of the original and processed image sequences used in this figure was trimmed to contain only two contraction–relaxation cycles. The top left panel shows a maximum intensity projection of the original, unprocessed sequence. The motion artifacts are seen as blurring and smearing of the cell outlines. A pseudo line-scan image in the middle left panel obtained by re-slicing of the original image sequence along the line shown in the top panel indicates that movement of the cells was associated with the global [Ca^2+^]_i_ transients occurring in all cells at the same time. As illustrated in the bottom left panel in Fig. 1, motion artifacts distort the time course of [Ca^2+^]_i_ traces making it difficult to obtain reliable single cell data. In most cases, application of the image processing algorithm eliminated these artifacts almost completely, thus allowing single-cell data to be extracted from the image sequences.

**Fig. 2:**
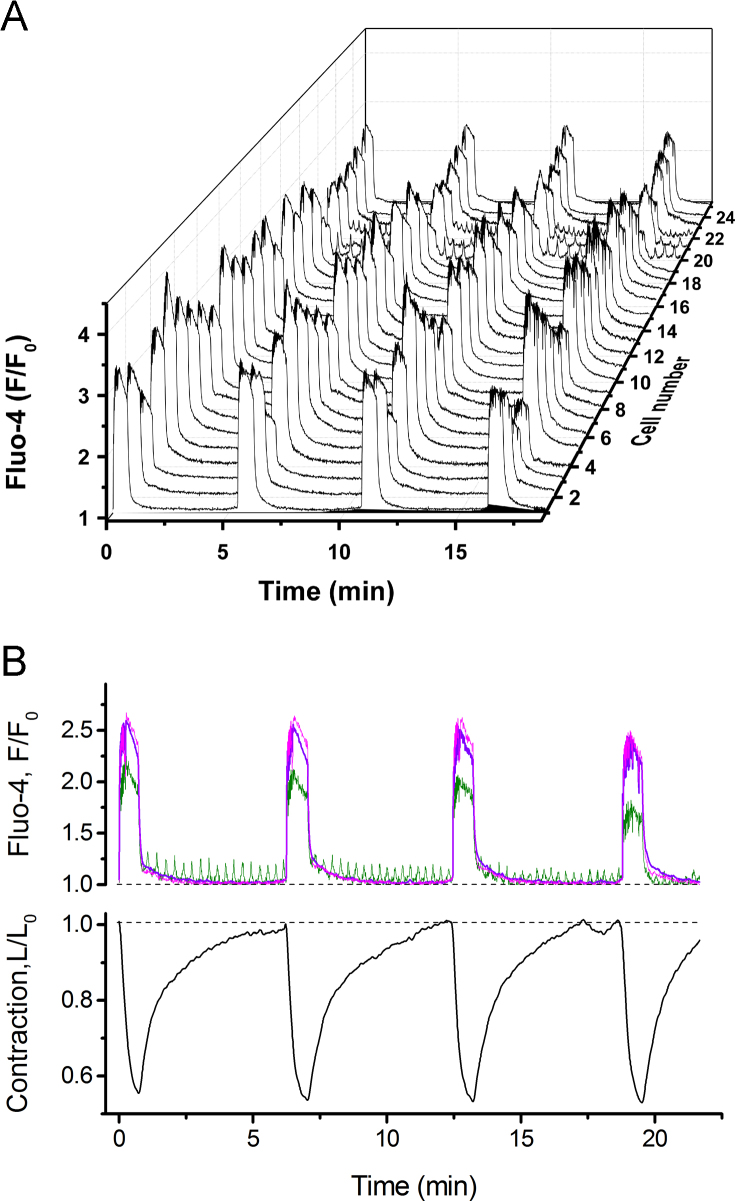
Single cell [Ca^2+^]_i_ transients in spontaneously contracting human myometrium. A. – a “3D waterfall” plot of [Ca^2+^]_i_ transients recorded from 25 cells during four consecutive contraction–relaxation cycles (representative of six experiments). B, upper panel – averaged [Ca^2+^]_i_ curve (magenta trace, average of all cells in A) superimposed with traces from an oscillating cell (green trace corresponding to cell 22 in A) and non-oscillating cell (violet trace corresponding to cell 23 in A). Lower panel in B shows slice displacement curve reflecting contractile activity. (For interpretation of the references to colour in this figure legend, the reader is referred to the web version of this article.)

Having validated the motion correction procedure, we investigated the single-cell [Ca^2+^]_i_ dynamics in spontaneously contracting slices of human myometrium (Fig. 2). In agreement with previously published data (Bru-Mercier et al., 2012), we found that all cells within any particular field of view were involved in the generation of global [Ca^2+^]_i_ transients accompanied by contractions (illustrated in Fig. 2B). While most of the cells were quiescent between global [Ca^2+^]_i_ transients, a small proportion of cells produced low-amplitude [Ca^2+^]_i_ oscillations that did not propagate to the neighbouring cells.

As shown in Fig. 2B, only global [Ca^2+^]_i_ transients triggered contractions, while [Ca^2+^]_i_ oscillations between the global events had no visible impact on mechanical activity of the tissue. There was more cell-to-cell variation in the amplitude of [Ca^2+^]_i_ transients than in the duration measured as full width at half magnitude (FWHM) (Fig. 2). Interestingly, statistical comparison (ANOVA test) revealed that FWHM varied significantly between slices from different patients: 38.88±0.57 s; 33.25±0.76 s; 48.67±0.41 s and 37.11±1.05 s (*p*<0.05, 98 cells from 4 patients) while variation in mean amplitude was not significant.

**Fig. 3:**
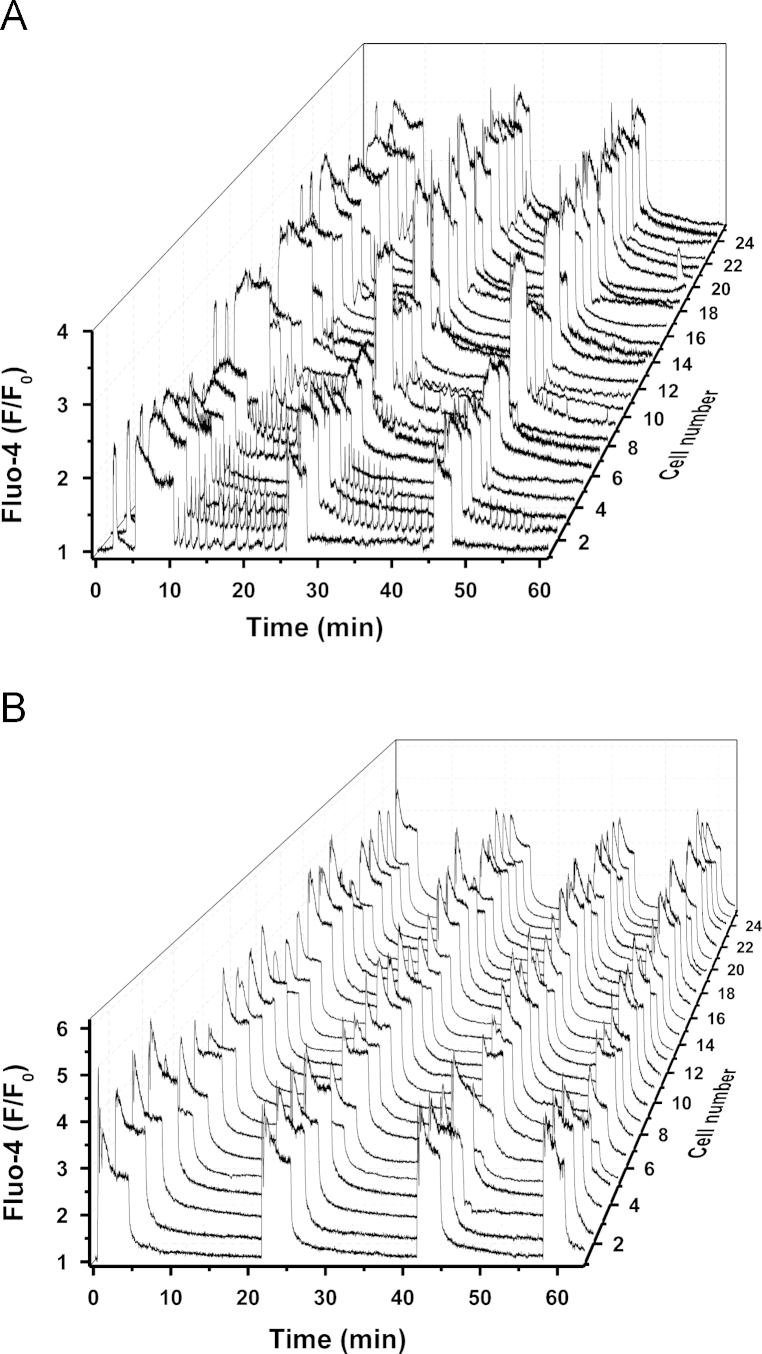
A – Immediate effects of oxytocin on [Ca^2+^]_i_ in myometrial slice: increase in duration and amplitude of the global [Ca^2+^]_i_ transients and induction of [Ca^2+^]_i_ oscillations in some cells; application of 10 nM oxytocin started at 5 min and was maintained for two hours. B – Established effect of oxytocin: augmentation of global [Ca^2+^]_i_ transients and cessation of [Ca^2+^]_i_ oscillations; same slice incubated with 10 nM oxytocin for 1 h.

Application of 10 nM oxytocin greatly increased the amplitude and duration of the global [Ca^2+^]_i_ transients in all cells tested. Oxytocin also elicited low-amplitude [Ca^2+^]_i_ oscillations in many but not all cells (Fig. 3A). Similar to unstimulated tissue, the oscillations occurred between global rises in [Ca^2+^]_i_ but they had a higher amplitude and were present in a larger number of cells.

**Fig. 4:**
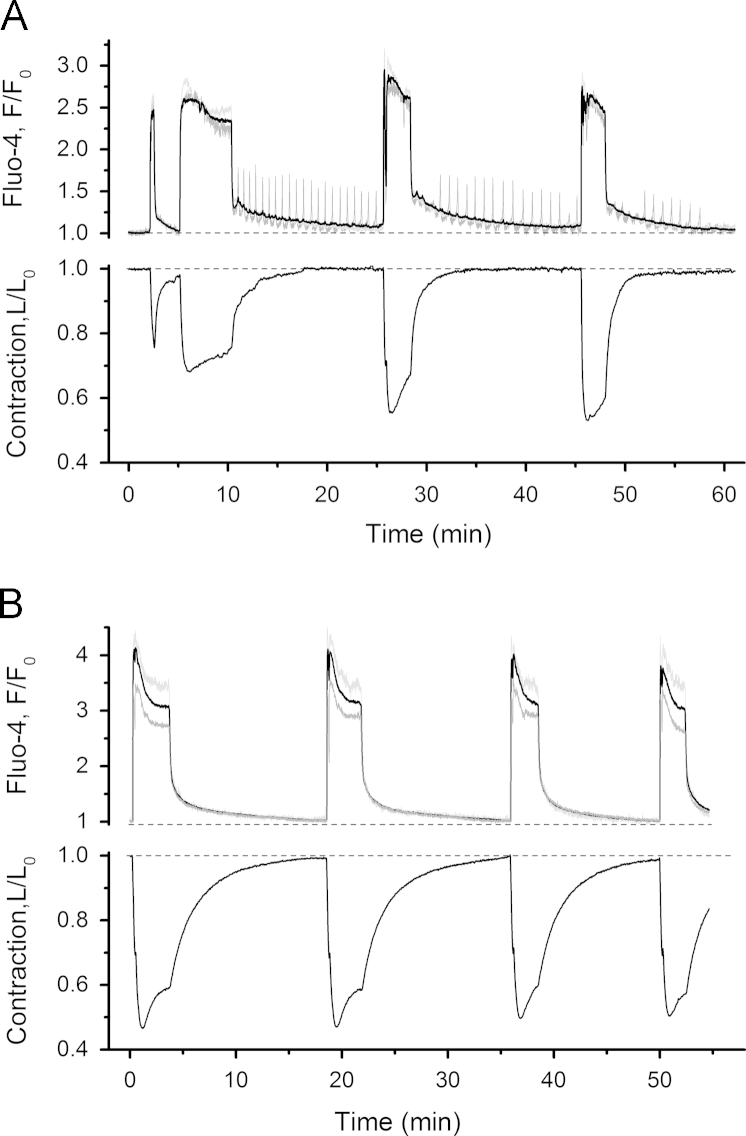
A – Oxytocin-induced [Ca^2+^]_i_ oscillations in individual cells are short-lived and disappear after several contraction–relaxation cycles. B – Potentiation of global [Ca^2+^]_i_ transients and phasic contractions in the presence of oxytocin is long-lasting. Upper panels in A and B – averaged [Ca^2+^]_i_ curve (thick black trace, average of all cells in Fig. 3) superimposed with traces from an oscillating cell (grey trace corresponding to cell 2 in Fig. 3) and non-oscillating cell (light grey trace corresponding to cell 16 in Fig. 3). Bottom panels in A and B show slice displacement curves reflecting contractile activity. Note slower rate of relaxation of phasic contractions in B compared to A.

While global [Ca^2+^]_i_ transients continued to increase in their amplitude and duration from cycle to cycle in the presence of oxytocin, the individual-cell [Ca^2+^]_i_ oscillations disappeared after 2–3 cycles. After 1 h treatment with oxytocin, the amplitude and duration of global [Ca^2+^]_i_ transients was increased by 127% and 683% respectively. Fig. 4A illustrates that, although present in a larger number of cells, the individual-cell [Ca^2+^]_i_ oscillations were not accompanied by any measurable contractions. That is, only augmented phasic contractions were observed in the presence of oxytocin. The rate of relaxation of phasic contractions was substantially lower after prolonged incubation with oxytocin compared to that at the beginning of oxytocin application. In summary, the sequence of events induced by oxytocin was as follows. Immediately after the application of oxytocin, a global [Ca^2+^]_i_ transient of dramatically increased duration and slightly potentiated amplitude was initiated. That was followed by induction of [Ca^2+^]_i_ oscillations in a subset of cells and by further progressive increase in the amplitude of global [Ca^2+^]_i_ transients and phasic contractions. After 2–3 contraction–relaxation cycles, the [Ca^2+^]_i_ oscillations in individual cells have subsided (see Fig. 4A) and a substantial decrease in the relaxation rate of high-amplitude phasic contractions was established (Fig. 4B).

## 4 Discussion

Elucidation of cellular mechanisms regulating myometrial contractility is crucial for improvement in management of many obstetric abnormalities, such as premature delivery, uterine dystocia and post-partum haemorrhage. Oxytocin is widely used by obstetricians for labour induction and for prevention of post-partum haemorrhage (Blanks, 2003; Wedisinghe et al., 2008). The physiological mechanism(s) mediating the effect of this hormone on uterine contractility are complex and remain incompletely understood (Fomin et al., 2009; Shmygol et al., 2006; Wray et al., 2003). As oxytocin receptors are known to activate the production of inositol-trisphosphate and subsequent release of Ca^2+^ from the sarcoplasmic reticulum, it is logical to expect that this process might be involved in the oxytocin-induced potentiation of myometrial contractility. Indeed, oxytocin-induced [Ca^2+^]_i_ oscillations have been demonstrated in primary cultures of human uterine myocytes (Fu et al., 2000; Young and Hession, 1996; Young and Zhang, 2001). However, to date, the oxytocin-induced [Ca^2+^]_i_ oscillations in intact cells residing within the myometrium have not been demonstrated due to methodological limitations associated with [Ca^2+^]_i_ imaging in contractile multicellular preparations. In the present study, we managed to overcome these limitations by combining confocal [Ca^2+^]_i_ imaging and advanced image processing to investigate the dynamics [Ca^2+^]_i_ on a cellular level in control and oxytocin stimulated slices of human myometrium. For the first time, we present evidence that oxytocin induces asynchronous [Ca^2+^]_i_ oscillations in individual myocytes within intact myometrium which are similar to those observed in cultured cells. Surprisingly, these oscillations seem to be unrelated to the oxytocin-induced augmentation of myometrial contractions and the tissue-level [Ca^2+^]_i_ transients as they occur only in a proportion of cells and quickly disappear, while augmentation of contractility in the presence of oxytocin persists for much longer. Our findings are compatible with the idea that myometrial contractions are regulated by the membrane oscillator (Berridge, 2008), likely, via the modulation of calcium and potassium channels and via changes in the Ca^2+^ sensitivity of contractile machinery. Based on the data obtained in this study, we conclude that oxytocin-induced [Ca^2+^]_i_ oscillations are not relevant to the acute regulation of myometrial contractility but may play a role in longer-term regulatory processes, for example, by triggering gene expression. In the present study, we did not investigate the effects of stretch on [Ca^2+^]_i_ and contractile responses to oxytocin. Undoubtedly, mechanical stretch is an important variable, especially at the end of pregnancy and during labour. More research on intact myometrium is needed to elucidate the effects of mechanical stretch on myometrial contractility and gene expression.

## Conflict of interest statement

The authors declare that they have no conflict of interest in regard to the above manuscript.
